# TAZ Induces Migration of Microglia and Promotes Neurological Recovery After Spinal Cord Injury

**DOI:** 10.3389/fphar.2022.938416

**Published:** 2022-06-27

**Authors:** Xuyang Hu, Jinxin Huang, Yiteng Li, Lei Dong, Yihao Chen, Fangru Ouyang, Jianjian Li, Ziyu Li, Juehua Jing, Li Cheng

**Affiliations:** Department of Orthopaedics, The Second Hospital of Anhui Medical University, Hefei, China

**Keywords:** TAZ, Fascin-1, microglia, migration, spinal cord injury

## Abstract

Following spinal cord injury (SCI), microglia gradually migrate to the edge of the lesion, interweaving around the border of the lesion to form the microglial scar, which performs inflammatory limiting and neuroprotective functions. Recent reports showed that Yes-associated protein (YAP) was expressed in astrocytes and promoted the formation of astrocytic scars, while YAP was not expressed in microglia after SCI. YAP and its paralogue transcriptional coactivator with PDZ-binding motif (TAZ) are transcriptional coactivators, which have a similar functional role as both are negatively regulated by the Hippo signalling pathway. However, the expression and function of TAZ after SCI are unclear. Our research group previously found that Fascin-1 was highly expressed in microglia and promoted migration of microglia after SCI, and that, there was a close regulatory relationship between Fascin-1 and YAP/TAZ. In this study, we demonstrated that TAZ was significantly upregulated and mainly expressed in microglia after SCI, and accumulated in the nuclei of microglia in the spinal cord at 14 days post-SCI. Moreover, TAZ was upregulated and accumulated in the nuclei of anti-inflammatory M2-like (M2-L) polarized or myelin-treated microglia. Additionally, XMU-MP-1 (an inhibitor of the Hippo kinase MST1/2 to active TAZ) promoted the aggregation of microglia around the lesion core, resulting in the formation of microglial scars and the functional recovery of mice after SCI. Our findings also indicated that TAZ promoted microglial migration *in vitro*. Mechanistically, Fascin-1 interacted with TAZ, which upregulated TAZ expression and induced TAZ nuclear accumulation in microglia to promote microglial migration. These findings revealed that TAZ mediated microglial migration to the edge of the lesion core, promoting the formation of microglial scars and functional recovery after SCI. Moreover, TAZ was downstream of Fascin-1, which positively regulated microglial migration after SCI.

## Introduction

Spinal cord injury (SCI) is a destructive neurological disease that causes loss of motor and sensory functions (Wang, Botchway et al., 2021). The pathophysiology of SCI is related to complicated molecular and cellular biological processes, that can be split into two phases: primary and secondary damage. The primary damage is generated by the initial injury to the spinal cord, which directly results in a cascade of secondary damages that lasts several weeks until the incision closes and the glial scar forms (Tran, Warren et al., 2018). This damage can cause a complex series of inflammatory and pathological processes that eventually lead to scarring. Following SCI, the scars mostly consist of astrocytic scars, fibrotic scars and microglial scars. Microglia migrate around the margin of the lesion core, and encircle the injury centre to form the microglial scar at the interface between the fibrotic scar and the astrocytic scar, which contributes to decreasing axon contraction and neuronal death, reducing lesion size, and promoting functional recovery (Bellver-Landete, Bretheau et al., 2019). However, the mechanism by which microglia migrate around the lesion core to form microglial scars is still unclear.

Our previous research confirmed that Fascin-1 was highly expressed specifically in microglia and promoted microglial migration after SCI, but the downstream molecules of Fascin-1 that promote microglial migration were not clear (Yu, Cheng et al., 2021). Fascin-1 is a F-actin bundling protein, mainly located at the fascicle core, that enables cells to form pseudopod-like structures and thus increases cell migration (Sun, He et al., 2011). Recent reports suggested that Fascin-1 can regulate the activity of Yes-associated protein (YAP)/transcriptional coactivator with PDZ-binding motif (TAZ), and that YAP/TAZ is the transcriptional coactivator of the Hippo signalling pathway, which can mediate cell migration (Kang, Wang et al., 2018; Zhou, Shen et al., 2020; Pu, Huang et al., 2021). This suggested that YAP/TAZ may mediate the promigratory effect of Fascin-1 on microglia after SCI. YAP is elevaled and activated in astrocytes after SCI, whereas, YAP is not expressed in microglia after SCI (Xie, Shen et al., 2020). Therefore, Fascin-1 cannot facilitate microglial migration by acting on YAP after SCI*.* At present, it is not clear whether the cellular localization and function of TAZ follow SCI and whether TAZ mediates the promigratory effect of Fascin-1 on microglia after SCI.

In this study, we demonstrated that the TAZ protein was elevated and displayed nuclear distribution in microglia at 14 days after SCI. TAZ was upregulated and entered the nucleus in myelin-treated microglia or anti-inflammatory M2-like (M2-L) microglia. Additionally, we found that XMU-MP-1 (an inhibitor of the Hippo kinase MST1/2 to active TAZ) increased the formation of microglial scars and improved the functional recovery of SCI. TAZ promoted microglial migration *in vitro*. Finally, our data indicated that Fascin-1 interacted with TAZ, and facilitated the migration of microglia by upregulating TAZ and inducing the entry of TAZ into the nucleus of microglia after SCI, and that there may be Fascin-1-TAZ pathway regulated microglial migration after SCI. Targeted activation of the Fascin-1-TAZ pathway is a potential means of treating SCI.

## Materials and Methods

### Animals

All study protocols involving mice complied with the principles of the Animal Committee of Ethics of Anhui Medical University (Approval No: LLSC20160052). Seven-week-old female C57BL/6J mice (weighing 17–22 g) were acquired from the Experimental Animal Center of Anhui Medical University. All mice were raised in a temperature-controlled room (23 ± 2°C) on a 12 h light-dark cycle with free access to water and food.

### Spinal Cord Injury Model and Treatment

The mice were anaesthetized with pentobarbital, which was injected intraperitoneally with 50 mg/kg. The spinal cords were completely exposed at the level of the T10 site through laminectomy. The exposed spinal cords were clamped with modified forceps for 5 s from both sides (McDonough, Monterrubio et al., 2015). Then, the wound was sutured layer by layer with thread after disinfection of the incision. The mice with SCI received routine anti-infective care and assisted urination care every day during their recovery. The sham-operated control group underwent laminectomy without injury. As reported in a previous study, intraperitoneal injection of XMU-MP-1 may be able to cross the blood-brain barrier (Qu, Zhao et al., 2018). XMU-MP-1 (Y-100526, MedChemExpress), dissolved in DMSO, was injected intraperitoneally at a dose of 1 mg/kg administered every 2 days (Fan, He et al., 2016).

### Myelin Preparation

The purification of myelin debris from mouse brain tissue via sucrose density gradient centrifugation, was performed as previously reported (Norton and Poduslo 1973). Briefly, the brain tissues were homogenized in a 0.35 M sucrose solution. We isolated myelin debris from brain tissues via sucrose density gradient centrifugation, including ultracentrifugation in 0.85 M sucrose (100,000 g, 4°C, 40 min), resuspension in Tris-Cl buffer (100, 000 g, 4°C, 40 min), washing (200,000 g, 4°C, 15 min) and resuspension in 10 ml buffer. A final concentration of 1 mg/ml was used to stimulate cells in all tests.

### Cell Culture and Transfection

The BV-2 microglial cell line was provided by the BeNa Culture Collection (Beijing, China, Resource ID: BNCC337749). BV-2 cells were cultured in DMEM (HyClone/Thermo), supplemented with 10% foetal bovine serum (FBS, Thermo -Fisher Scientific) at 5% CO_2_ and 37°C in a humidified atmosphere.

BV-2 cells were seeded in 6-well plates or 24-well plates at approximately 60%–70% confluency. Following the manufacturer’s transfection protocol, three different siRNAs targeting TAZ mRNA (siTAZ#1, siTAZ#2, siTAZ#3) were transfected into BV-2 cells using jetPRIM (Polyplus Transfection, 114–15). The sequences of siTAZ#1, siTAZ#2, and siTAZ#3 were as follows: siTAZ#1 sense (5′-CCC​UCU​UCA​ACU​CUG​UCA​UTT-3′), siTAZ#2 sense (5′-GCC​AGA​GAU​ACU​UCC​UUA​ATT-3′), and siTAZ#3 sense (5′-CUG​CCU​UCC​AGG​AAC​AAA​UTT-3′). Three different siRNA targeting Fascin-1 mRNA (siFascin-1#1, siFascin-1#2, siFascin-1#3) were transfected into BV-2 cells through using jetPRIM (Polyplus Transfection, 114–15). The sequences of siFascin-1#1, siFascin-1#2, siFascin-1#3 were as follows: siFascin-1#1 sense (siRNA:5′-CAGCCAAUCAGGAUGAAGATT-3′), siFascin-1#2 sense (siRNA:5′-CCGCAGGUUAACAUCUACATT-3′), siFascin-1#3 sense (siRNA:5′-GAUGCCAACCGUUCCAGUUTT-3′). The siRNA and nonspecific control (siNC) were designed and synthesized by GenePharma (Shanghai, China). The TAZ overexpression plasmid pCMV-HA-TAZ (HA-TAZ), Fascin-1 overexpression plasmid pcDNA3.1-Flag-Fascin-1 (Flag-Fascin-1), and the empty vectors pCMV-HA (HA) and pcDNA3.1-Flag (Flag) were designed and synthesized by Shanghai Haoge Biotechnology Limited Company. The plasmids were transfected into BV-2 cells by Lipofectamine 2000 (Invitrogen). These cells were further cultured in DMEM for 24 h in 5% CO_2_ at 37°C and collected for subsequent experiments.

### Tissue Preparation

The mice wereanaesthetized with 0.9% normal saline and 4% paraformaldehyde (PFA), and a 6 mm portion of the spinal cord surrounding the injury centre was extracted. The tissues were placed into the dewatering tank for dehydration: 75% alcohol for 4 h, 85% alcohol for 2 h, 95% alcohol for 1 h, anhydrous ethanol twice, for 30 min each time, alcohol benzene for 5–10 min, xylene twice, for 5–10 min each time, and a wax soak for 3 h. After the dehydration process was complete, the wax-soaked tissue was embedded in the embedding machine. Then, the wax block was placed on the paraffin slicer to obtain 6 µM thick sagittal sections. During western blot analysis, the blood of mice was removed by 0.9% normal saline perfusion through the heart after anaesthesia, and spinal cord tissue 6 mm from the lesion centre was collected.

### Immunofluorescence Staining Analysis

The spinal cord sections were fixed with 4% PFA for 48 h at 4°C. The sections were dehydrated, cleared, wax-soaked, and paraffin-embedded. The sections were sliced into 6 µM thick sections with a microtome for drying, dewaxing, and antigen retrieval. Subsequently, the spinal cord tissue sections were blocked with 5% goat serum (Beyotime) −0.3% Triton X-100- phosphate buffered saline (PBS) at room temperature for 1 h. The sections were incubated overnight with the following primary antibodies at 4°C: mouse anti-TAZ (1:100, Proteintech, 66500-1-lg), rabbit anti-Fascin-1 (1:100, Abcam, ab126772), goat anti-Iba1 (1:100, Novus Biologicals, NB100-1028), rabbit anti-CX3CR1 (1:200, Abcam, ab8021), rabbit anti-GFAP (1:100, Proteintech, 16825-1-AP), and goat anti-PDGFRβ (1:100, R&D Systems, AF1042-SP). The sections were subsequently incubated with secondary antibodies for 1 h at room temperature in the dark. We used the secondary antibodies conjugated with Alexa Fluor 488(Invitrogen), and Alexa Fluor 594 (1:500, A-21202, A-21207, A-11058, Invitrogen). Finally, the tissues were stained with 4′,6-diamidino-2-phenylindole (DAPI 1:1000, Sigma Millipore) to visualize the nuclei. The images were acquired through a fluorescence microscope (Zeiss).

### Immunocytochemistry Staining

After BV-2 cells were cultured with different treatments, they were fixed in 4% PFA for 25 min. Then, they were blocked with 5% goat serum-0.3% Triton X-100- PBS at room temperature for 50 min. The cells were incubated overnight at 4°C with primary antibodies, the primary antibodies included mouse anti-TAZ (1:100, Proteintech, 66500-1-lg), rabbit anti-Fascin-1 (1:100, Abcam, ab126772). The next day, the cells were treated with secondary antibodies for 1 h at room temperature as outlined above. Finally, the cells were stained with DAPI (1:1000, Sigma Millipore) and the images were photographed using a fluorescence microscope (Zeiss).

### Microglial Polarization

BV-2 cells were cultured in 6-well plates or 24-well plates to approximately 70–80% confluence. After incubating in serum-free DMEM for 24 h, the cells were treated with lipopolysaccharide (LPS; 100 ng/ml, Beyotime Biotechnology, ST1470, Shanghai, China) plus IFNγ (20 ng/ml, Beyotime Biotechnology, China) for M1-L polarization, and IL-4 (20 ng/ml, Beyotime Biotechnology, P5916) for M2-L polarization, after which the BV-2 cells were cultured for 24 h.

### Western Blot Analysis

The spinal cords or cultured microglia were extracted using RIPA buffer (Solarbio Science & Technology Co., Ltd. Beijing, China), which included protease and phosphatase inhibitors. Next, equal amounts of protein samples were separated by 10% SDS-PAGE gels and subsequently blotted onto PVDF membranes. The membranes were blocked with 5% fat-free milk at room temperature for 1 h. Afterwards, the membranes were incubated with various primary antibodies, including mouse anti-GAPDH (1:5000, Proteintech, 60004-1-Ig), rabbit anti-Fascin-1 (1:300, Abcam, ab126772), rabbit anti-TAZ (1:500, Affinity, DF4653), mouse anti-TAZ (1:500, Proteintech, 66500-1-lg) at 4°C overnight. The membranes were incubated with goat anti-mouse (1:5000, A4416, Sigma, United States), and goat anti-rabbit (1:10000, 19003-1-AP, Proteintech) secondary antibodies for 2 h. GAPDH was used as an internal control. The protein bands were detected using enhanced chemiluminescence (ECL LI-COR Inc. United States). ImageJ was used for quantitative analysis.

### Transwell Assay

The migration ability of BV-2 cells was assessed by Transwell assays using Transwell chambers (Costar, 3415). Different treatment groups were designed according to the experimental requirements. Serum-free cell suspensions were seeded in the upper chamber (1×10^5^ cells per chamber), and the lower chamber was filled with 10% FBS for 24 h. The chamber cells were fixed with 4% PFA for 20 min and stained with crystal violet for 15 min, after washing with ddH_2_O water 3 times. The stained cells were observed and counted under an inverted microscope.

### Scratch Assay

BV-2 cells were seeded in 6-well plates until reaching approximately 90–100% confluence. Then, according to the different design groups, BV-2 cell monolayers in the control group and treatment group were scratched with 200 μl sterile pipets to form a wound-like gap. At 0 h, dead cells and floating cells were removed with PBS, after which the cell monolayer was cultured in DMEM. The migration of the cells was observed, and images were recorded at 0 h and 24 h. The wound healing closure rate was analysed using ImageJ.

### Coimmunoprecipitation

The relationship between Fascin-1 and TAZ in 7-day SCI tissues and myelin-treated microglia was assessed by coimmunoprecipitation (Co-IP) assay. The 7-day SCI tissues and myelin-treated microglia were lysed using Co-IP lysis buffer containing protease/phosphate inhibitors (P1050, Beyotime Biotechnology, Shanghai, China) for 30 min. The supernatants were extracted after centrifugation at 12,000 g for 30 min at 4°C. The supernatants were incubated with the primary antibodies, an anti-Fascin-1 antibody or IgG overnight at 4°C. After the immunoprecipitation reaction, the immunoprecipitated protein was mixed with protein A/G beads, and the mixture was incubated for 3 h at 4°C. The beads were washed three times with ice-cold washing buffer, and then eluted by heating in 2 × SDS loading buffer for 10 min. The proteins were collected for subsequent immunoblotting of Fascin-1 and TAZ.

### Behavioural Assessment

The mice were tested for recovery of locomotor function 0, 3, 7, 14, and 28 days after SCI via the Basso Mouse Scale (BMS). This rating scale evaluated not only trunk stability, but also limb movement, stepping, and coordination in an open field. The scale ranges from 0 (completely paralyzed) to 9 (completely normal) (Basso, Fisher et al., 2006). Footprint analysis was used to assess the locomotor capacity of the front and back limbs at 28 days after SCI, the forelimbs and hind limbs were immersed in green and red dyes, and the mice walked on a narrow track lined with white paper to measure paw rotation, stride length and width (Ma, Basso et al., 2001). The behavioural tests were performed independently by four reviewers who were blinded to the groups of mice.

### Statistical Analysis

All experiments were carried out independently, and GraphPad Prism software (version 8.0, GraphPad Software, USA) was used for statistical analysis. The results are expressed as the means ± standard error of the mean (SEM), and multiple-group comparisons were statistically analysed by one-way analysis of variance (ANOVA) with Tukey’s method. *p* <0.05 suggested a significant difference.

## Results

### Transcriptional Coactivator With PDZ-Binding Motif is Upregulated After Spinal Cord Injury, Primarily in Iba1^+^ Cells Around the Lesion Core

**FIGURE 1:**
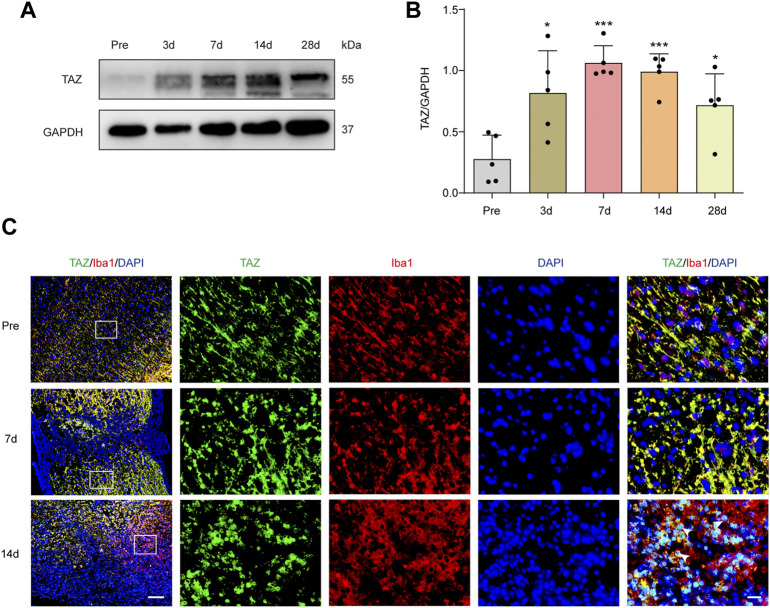
TAZ was significantly increased and localized in microglia. **(A)** Western blot analysis of TAZ expression in spinal cords at 3d, 7d, 14d, and 28d after SCI, compared with Pre (pre-operation, Pre). **(B)** Quantitative analysis of TAZ level as shown in (A). The blots (*n* = 3 per group) were quantified by a densitometric method using ImageJ software. GAPDH was used as the loading control. Data were mean ± SEM. *p<0.01(Pre vs. 3d or 28d); ***p<0.001 (Pre vs. 7d or 14d). **(C)** Immunofluorescence labeling in sagittal section of spinal cords showing the spatiotemporal distribution of Iba1 (red), TAZ (green), and DAPI (blue) at Pre, 7d and 14d after SCI. White arrows indicated that TAZ aggregated in the nuclei of Iba1^+^ cells. The asterisks indicated the centre of the lesion. Scale bars: low magnification, 100 μM; high magnification, 20 μM.

**FIGURE 2:**
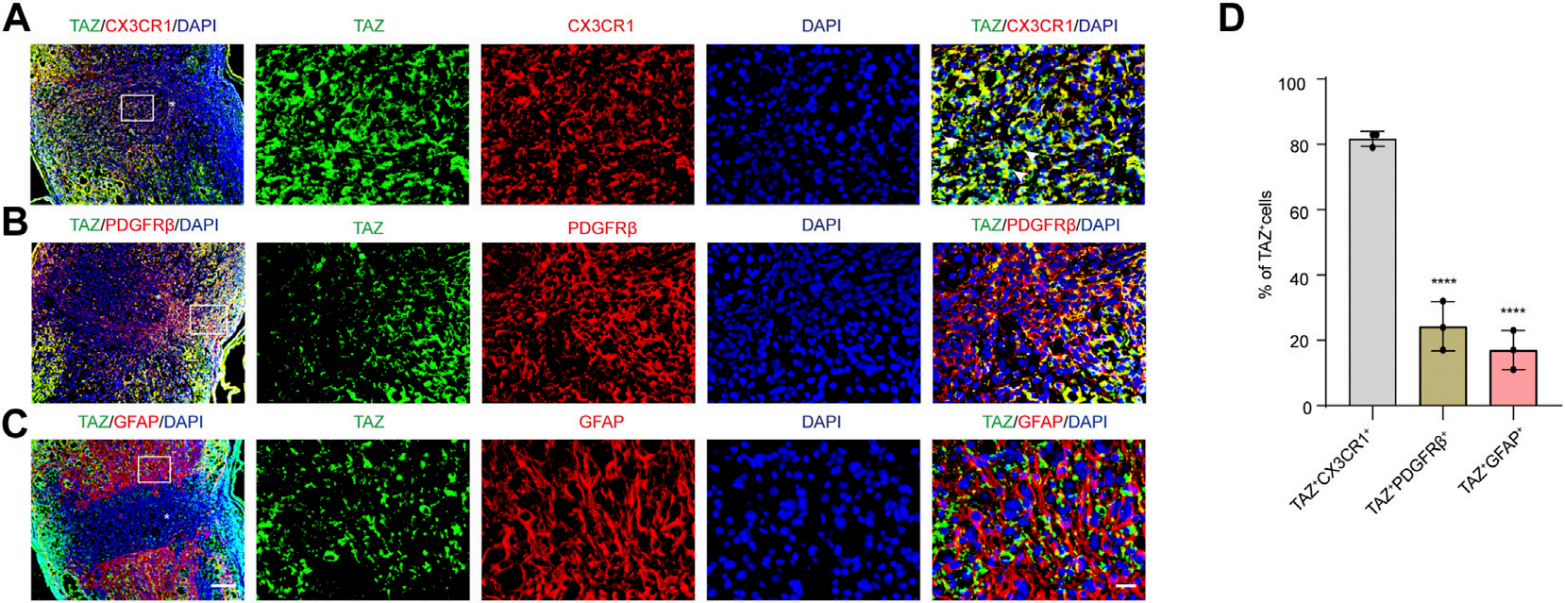
The expression pattern of TAZ in spinal cord tissues at 14 days after SCI. **(A–C)** Double immunostaining of TAZ (green) and CX3CR1 (red) **(A)**, TAZ (green) and PDGFRβ (red) **(B)**, or TAZ (green) and GFAP (red) **(C)** in spinal cords at 14 days after SCI. White arrows indicated that TAZ aggregated in the nuclei of CX3CR1^+^ cells **(D)** Quantitative analysis of the percentage of TAZ^+^CX3CR1^+^, TAZ^+^PDGFRβ^+^ and TAZ^+^GFAP^+^ cells in total TAZ^+^ cells in injured spinal cords. Scale bars: low magnification, 100 μM; higher magnification, 20 μM. The asterisks indicated the centre of the lesion. Data were mean ± SEM (*n* = 3 independent experiments). ****p<0.0001 (TAZ^+^PDGFRβ^+^ or TAZ^+^GFAP^+^ vs. TAZ^+^CX3CR1^+^).

To explore the changes in TAZ expression at different stages after SCI, western blotting was performed, and the results suggested that the expression of TAZ protein was notably upregulated at the lesion site from 3 days and reached its peak at 7 days following SCI in comparison to that before injury (Figures 1A,B). Iba1 is a common marker of microglia, that can be used to identify microglia after SCI (McDonough, Hoang et al., 2013; Ullah, Choe et al., 2018). To explore the temporal and spatial expression pattern of TAZ in the tissues on different days after SCI, we performed double immunostaining of TAZ and Iba1. The results showed that compared with before SCI, TAZ was increased significantly at 7 and 14 days after SCI. TAZ and Iba1 were predominantly colocalized around the injury core, which was corresponded to the localization of microglia after SCI. In addition, the TAZ protein was expressed in the cytoplasm at 7 days after SCI, and displayed the nuclear distribution in Iba1^+^ microglia at 14 days after SCI (Figure 1C). After SCI, the spinal cord tissue is injured, and activated astrocytes, microglia and pericytes together form a dense border to isolate the severely injured region (Tran, Warren et al., 2018). To further analyse the spatial expression pattern of TAZ after SCI, we performed double immunostaining with TAZ and several cell markers, including CX3CR1 (microglia marker), PDGFRβ (pericytes marker) and GFAP (astrocytes marker), in the mouse spinal cords. Our data suggested that TAZ was highly expressed in CX3CR1^**+**^ microglia, and slightly expressed in GFAP^+^ astrocytes or PDGFRβ^+^ pericytes around the lesion core. Moreover, TAZ protein exhibited nuclear distribution in CX3CR1^+^ microglia 14 days after SCI (Figure 2). In summary, these results indicated that TAZ was significantly upregulated after SCI, principally surrounded the injury core, and its expression pattern was similar to that of microglia, implying that TAZ may be involved in the formation of microglial scars after SCI.

### Transcriptional Coactivator With PDZ-Binding Motif Upregulates and Accumulates in the Nuclei in M2-L or Myelin-Treated Microglia

**FIGURE 3:**
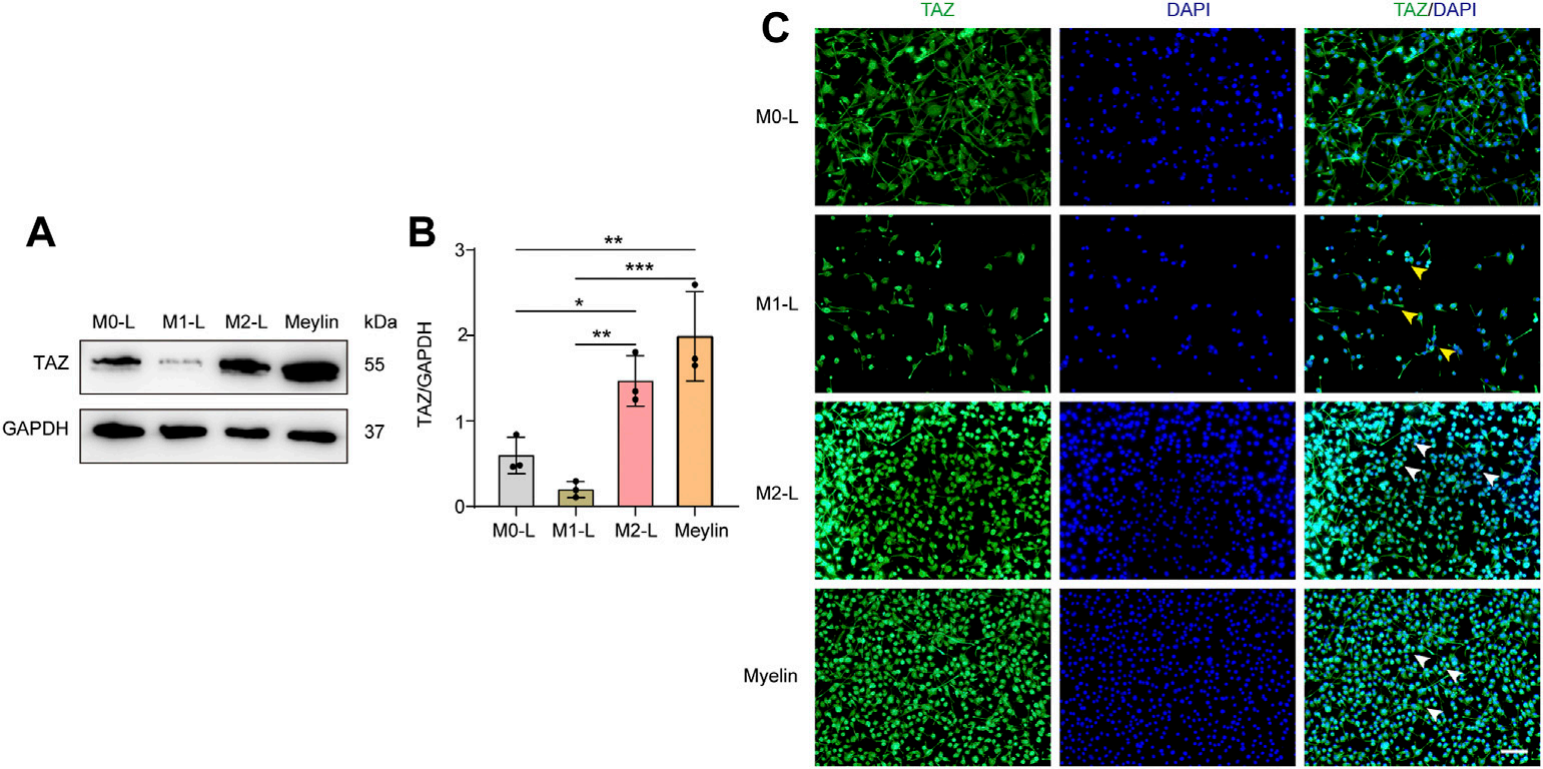
The changes of TAZ after the polarization of microglia and myelin treatment. **(A)** Western blot was used to detect the changes of TAZ expression in the different microglial phenotypes and myelin treatment (*n* = 3 per group), microglia were differentiated into pro-inflammatory M1-like (M1-L), anti-inflammatory M2-like (M2-L), and resting, unstimulated (M0-L) phenotypes. **(B)** Quantitative analysis of TAZ expression in **(A)**. GAPDH was used as the loading control. Data were mean ± SEM. *p<0.05 (M0-L vs. M2-L); **p<0.01 (M1-L vs. M2-L); **p<0.01 (M0-L vs. Meylin); ***p<0.001 (M1-L vs. Meylin). **(C)** Representative immunofluorescence images of TAZ (green) in M0-L, M1-L, M2-L microglia and myelin-treated microglia, and the nuclei were stained with DAPI (blue). Yellow arrows indicated that TAZ gathered around the nuclei of microglia. White arrows indicated that TAZ accumulated the nuclei of microglia. Scale bars: low magnification, 100 μM; higher magnification, 20 μM.

According to relevant studies, microglial polarization is a critical event in secondary damage after SCI, and the aggregation of microglia at the damage core plays a vital role in the inflammatory response to secondary injury (Xu, Wang et al., 2021). Microglia can be polarized into proinflammatory M1-like (M1-L), anti-inflammatory M2-like (M2-L), and resting, unstimulated (M0-L) phenotypes, which participate in the regulation of the SCI microenvironment (Fu, Chen et al., 2022). In addition, some studies have shown that myelin uptake predisposes microglia/macrophages to immunosuppressive and neurotrophic phenotypes, and that the elimination of myelin fragments (phagocytosis) at the site of demyelination by microglia is crucial for remyelination in the central nervous system (Miron, Boyd et al., 2013). We further explored the changes in the expression and localization of TAZ in different microglial states by simulating the *in vivo* microenvironment of SCI *in vitro*, through BV-2 microglia cultured to induce microglial polarization and with myelin-treated microglia. Western blot results showed that compared with that in M0-L microglia, TAZ expression was downregulated in M1-L microglia, whereas, TAZ expression was upregulated in M2-L or myelin-treated microglia (Figures 3A,B). Furthermore, analysis of TAZ by immunofluorescence staining revealed that TAZ accumulated around the nucleus in M1-L microglia, nevertheless, TAZ accumulated in the nucleus of M2-L microglia or myelin-treated microglia (Figure 3C). These results suggested that TAZ was upregulated and entered the nucleus in myelin-treated or M2-L microglia.

### Activation of Transcriptional Coactivator With PDZ-Binding Motif Signalling by XMU-MP-1 Promotes the Formation of Microglial Scar and Improves Functional Recovery after Spinal Cord Injury

**FIGURE 4:**
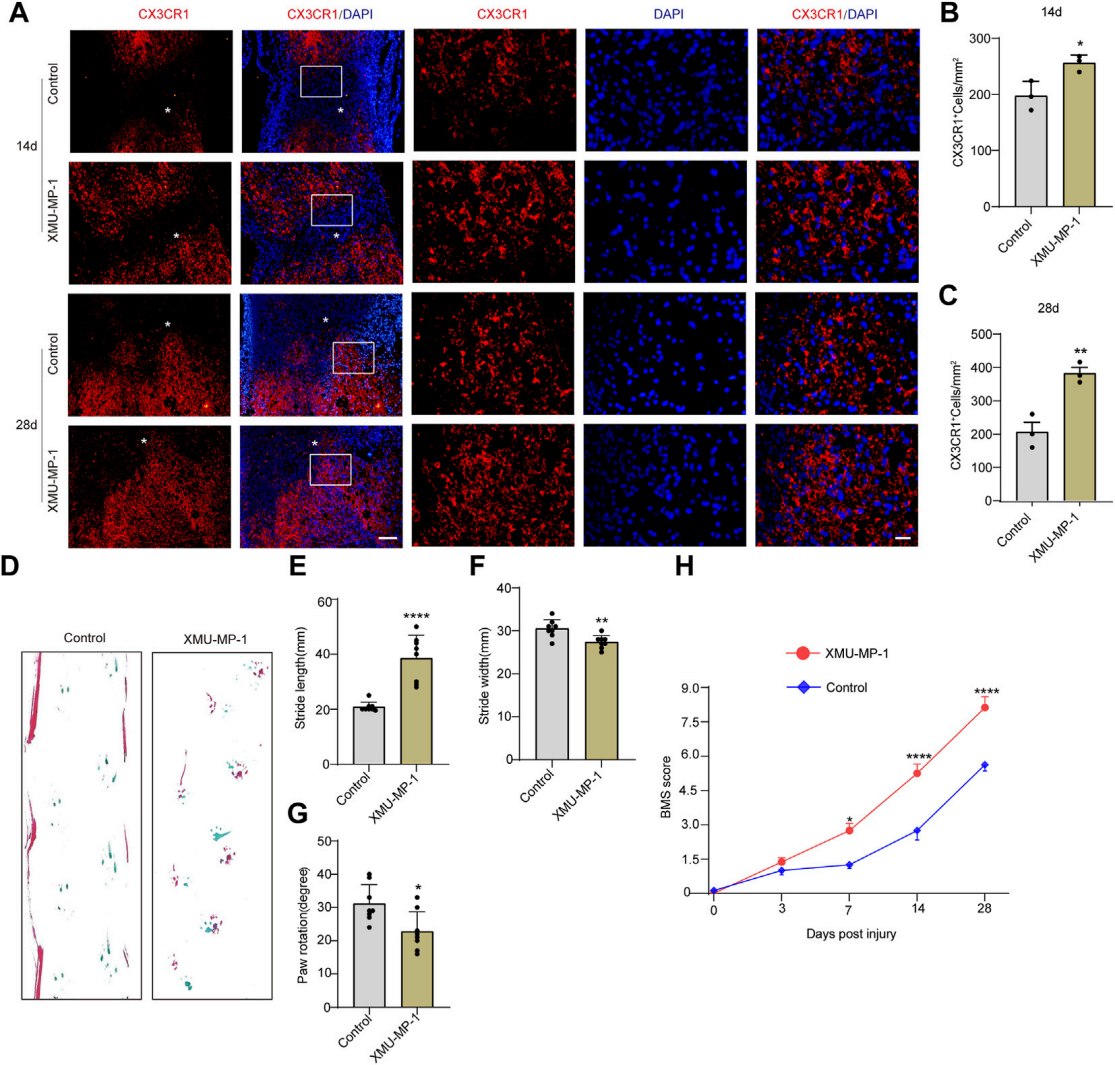
Activation of TAZ by XMU-MP-1 promoted the formation of microglial scars and functional recovery after SCI. **(A)** Immunostaining of CX3CR1 (red) in the spinal cords of control and XMU-MP-1 treated mice at 14, 28 days after SCI (*n* = 3 per group). **(B, C)** Quantitative analysis of the number of CX3CR1^**+**^ microglia per mm^2^ as shown in (A). Data were mean ± SEM. *p<0.05 (control vs. XMU-MP-1) in (B, C). Scale bars: low magnification, 100 μM; higher magnification, 20 μM. **(D)** Representative the footprint analyses treated with XMU-MP-1 and control group at 28 days after SCI (*n* = 8 per group). (**E–G)** Quantitative data were analyzed the grip walk tests treated with XMU-MP-1 and control group at 28 days after SCI in (D). The asterisks indicated the centre of the lesion. Data were mean ± SEM. ****p<0.0001, **p<0.01, *p<0.05, compared with control group. **(H)** The time course of the functional recovery based on the Basso Mouse Scale score treated with XMU-MP-1 and control group after SCI (*n* = 8 per group).

Based on the high expression and activation of TAZ in microglia after SCI, we further examined the functions exerted by TAZ after SCI. Recent studies have suggested that XMU-MP-1 can inhibit mammalian STE20-like protein kinase 1 (MST1) and mammalian STE20-like protein kinase 2 (MST2) to facilitate their downstream TAZ activation. Next, we tested whether XMU-MP-1 could promote the formation of microglial scars and functional recovery after SCI. Immunostaining results showed that compared with the control group, the formation of microglial scars was notably increased following XMU-MP-1 therapy (Figures 4A–C). Furthermore, the footprint analysis and BMS score revealed that XMU-MP-1 notably promoted functional recovery in mice following SCI in comparison to the control treatment (Figures 4D–H). These results showed that activation of TAZ facilitated the migration and accumulation of microglia to form microglial scars at the lesion border and improve the functional recovery of mice after SCI.

### Transcriptional Coactivator With PDZ-Binding Motif Promotes Microglial Migration *in Vitro*

**FIGURE 5:**
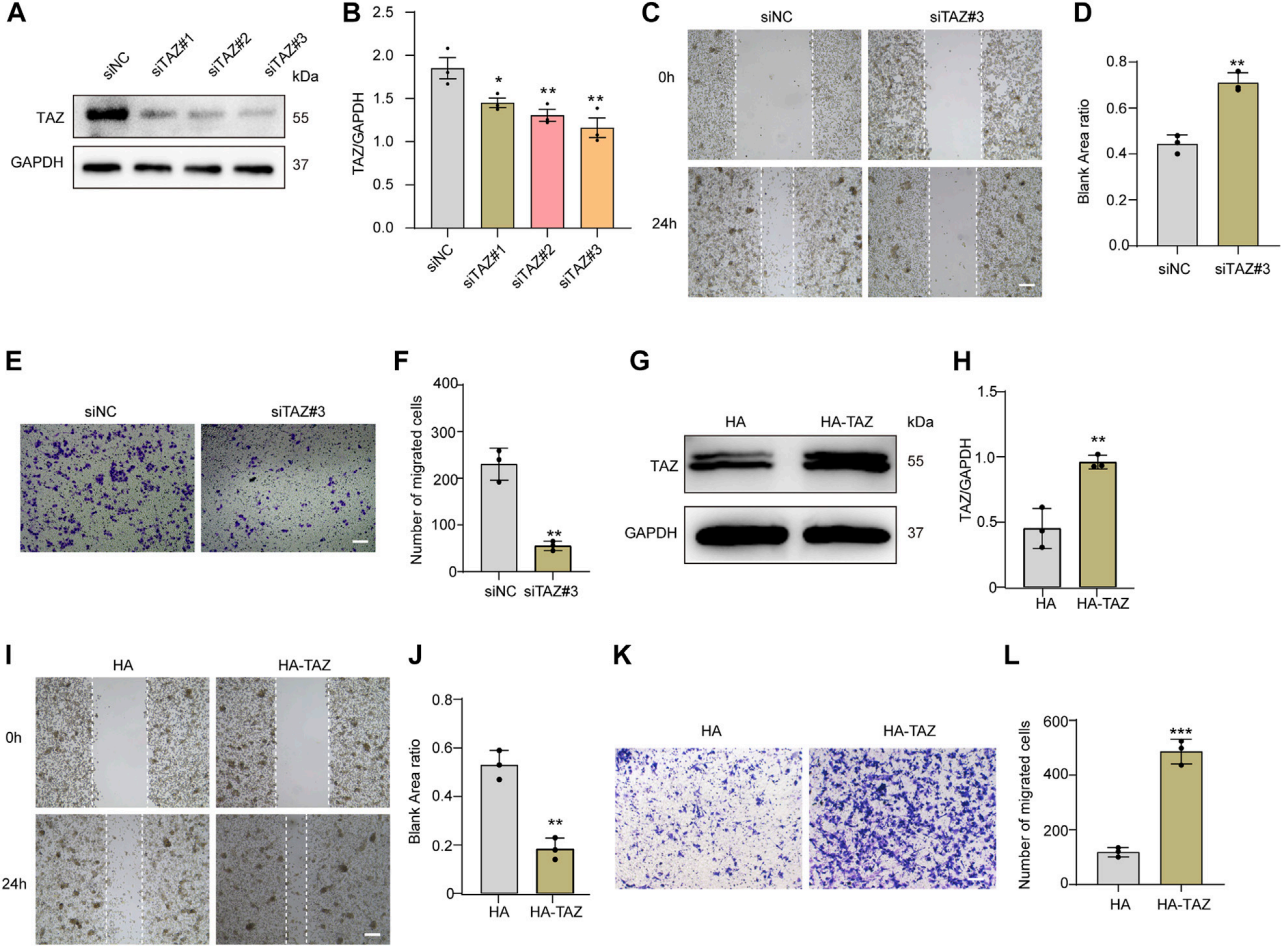
TAZ promoted microglial migration *in vitro*. **(A)** Western blot analysis of the expression of TAZ was detected after transfection with siTAZ (knockdown) and siNC (control). **(B)** Quantitative analysis of TAZ expression in (A). GAPDH was used as the loading control. The blots (*n* = 3 per group) were quantified as previously described. Data were mean ± SEM. *p<0.05 (siNC vs. siTAZ#1); **p<0.01 (siNC vs. siTAZ#2 or siTAZ#3). According to the results, siTAZ#3 knockdown was the best effect on transfection, and siTAZ#3 was selected for transfection. **(C,E)** For the scratch assay and Transwell analysis were used to detect the migration of microglia after transfection with siNC and siTAZ#3 for 24 h, and cell migration was recorded 0 and 24 h (*n* = 3 per group). Scale bar: 200 μM. **(D)** Quantification of blank area ratio in (C). Data were mean ± SEM. **p<0.01 (siNC vs. siTAZ). **(F)** Quantitative analysis of the number of transmembrane cells in **(E)**. Data were mean ± SEM. **p<0.01 (siNC vs. siTAZ). Scale bars: 200 μM. **(G)** Western blot analysis of the expression of TAZ was detected after plasmid HA-TAZ (overexpression) and HA (control) were transfected into microglia. **(H)** Quantitative analysis of TAZ expression in **(G)**. The imprints (*n* = 3 per group) were quantified by densitometry using ImageJ software. Data were mean ± SEM. **p<0.01 (HA vs. HA-TAZ). **(I,K)** Scratch and Transwell analysis, which were used to detect microglial migration after transfection with plasmid HA-TAZ and HA into microglia for 24 h (*n* = 3 per group), cell migration was recorded 0 and 24 h. Scale bar: 200 μM. (J) Quantification of blank area ratio in (I). Data were mean ± SEM. **p<0.01 (HA vs. HA-TAZ). **(L)** Quantitative analysis of the number of transmembrane cells in **(K)**. Data were mean ± SEM. **p<0.01 (HA vs. HA-TAZ). Scale bars: 200 μM.

Given that the migration of microglia to the lesion core after SCI is the key process of microglial scar formation, and that TAZ can also promote cell migration, we next examined whether TAZ was required for the migration of microglia *in vitro*. The TAZ knockdown model was constructed by three different siRNAs targeting TAZ mRNA (siTAZ#1, siTAZ#2, siTAZ#3), of which siTAZ#3 was determined to have the optimal knockdown efficiency as evaluated by Western blot, and therefore siTAZ#3 was used in the subsequent experiments (Figures 5A,B). Scratch tests were used to investigate the effect of TAZ knockdown on microglial migration. The results showed that the healing rate of the siTAZ#3 group was significantly less than that of the siNC group (Figures 5C,D). In addition, the Transwell assays were performed to explore the effect of TAZ knockdown on the migration of microglia. The number of cells crossing the lower chamber in the siTAZ#3 group was less than in the siNC group, which was consistent with the scratch assay results (Figures 5E,F). This suggested that TAZ knockdown inhibited the migration ability of microglia. To further investigate the effect of TAZ on regulating microglial migration, we transfected the HA-tagged expression vector HA-TAZ into microglia to upregulate TAZ expression levels. Western blot results indicated upregulation of the TAZ protein in microglia transfected with HA-TAZ compared with those transfected with the empty HA vector (Figures 5G,H). Then, the scratch assays were conducted to assess the effect of TAZ overexpression on microglial migration. The findings revealed that the healing rate of the HA-TAZ group was notably higher than that of the HA group (Figures 5I,J). In addition, to explore the effect of TAZ overexpression on microglial migration, the Transwell tests were used. More cells crossed the lower chamber in the HA-TAZ group than in the HA group, which was consistent with the scratch assay results (Figures 5K,L). These results suggested that TAZ promoted migration of microglia.

### Fascin-1 Upregulates Transcriptional Coactivator With PDZ-Binding Motif and Mediates Transcriptional Coactivator With PDZ-Binding Motif Translocation Into the Nucleus *in Vitro*

**FIGURE 6:**
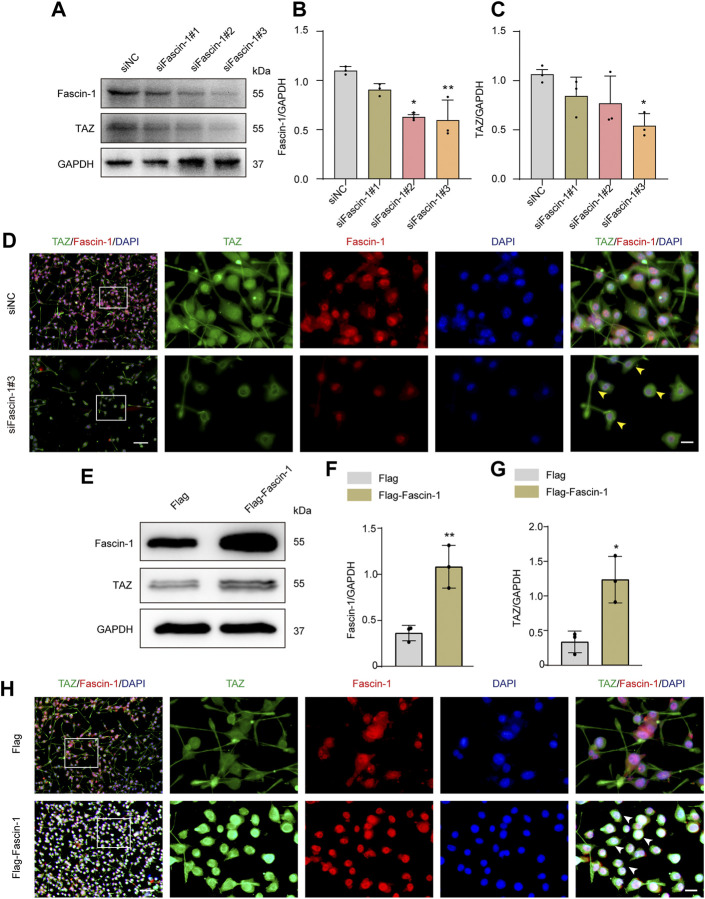
Fascin-1 upregulated TAZ and mediated TAZ into the nuclei of microglia *in vitro*. **(A)** Western blot was used to detect the expression levels of Fascin-1 and TAZ after transfection with siFascin-1 (knockdown) and siNC (control) (*n* = 3 per group). **(B,C)** Quantitative analysis of the relative levels of Fascin-1 (B) and TAZ (C) as shown in (A). Protein expression was normalized to GAPDH. Data were mean ± SEM. *p<0.05 (siNC vs. siFascin-1#2) in (B); **p<0.01 (siNC vs siFascin-1#3) in (B); *p<0.05 (siNC vs. siFascin-1#2 or siFascin-1#3) in (C). The results showed that knockdown siFascin-1#3 was the best effect on transfection. **(D)** After transfection of siFascin-1 and siNC into microglia *in vitro*, double immunostaining analysis of TAZ (green) and Fascin-1 (red) in microglia. Scale bars: low magnification, 100 μM; higher magnification, 20 μM. Yellow arrows indicated that TAZ gathered around the nuclei of microglia. **(E)** Western blot was used to exam the expression levels of Fascin-1 and TAZ after transfection with plasmid Flag-Fascin-1 (overexpression) and Flag (control) (*n* = 3 per group). **(F,G)** Quantitative analysis of the relative levels of Fascin-1 (F) and TAZ (G) as shown in (E). Data were mean ± SEM. **p<0.01 (Flag vs Flag-Fascin-1) in (F). *p<0.05 (Flag vs. Flag-Fascin-1). **(H)** Double immunostaining analysis of TAZ (green) and Fascin-1 (red) in microglia after transfection with Flag and Flag-Fascin-1. White arrows indicated that TAZ accumulated the nuclear of microglia. Scale bars: low magnification, 100 μM; higher magnification, 20 μM.

Our team previously revealed that Fascin-1 was highly expressed specifically in microglia and promoted microglial migration after SCI. Recent report indicated that Fascin-1 can mediate TAZ activity (Yu, Cheng et al., 2021). The above results suggested that TAZ was upregulated and accumulated in the nucleus in microglia after SCI. Moreover, TAZ was involved in microglial migration after SCI, and this process might be closely linked to the upregulation of Fascin-1 expression after SCI. Therefore, we explored the effect of Fascin-1 on the subcellular localization and expression of TAZ in microglia. The Fascin-1 knockdown model was constructed by three different siRNAs targeting Fascin-1 mRNA (siFascin-1#1, siFascin-1#2, siFascin-1#3). Western blot results confirmed that Fascin-1 knockdown notably inhibited Fascin-1 and TAZ expression in microglia, siFascin-1#3 showed the optimal knockdown efficiency as assessed by Western blotting and was therefore used for subsequent experiments (Figures 6A–C). To examine the effects of Fascin-1 on the subcellular localization of TAZ in microglia, immunofluorescence staining revealed that knockdown of Fascin-1 caused TAZ to accumulate around the nucleus in microglia compared with the control group (Figure 6D). This result indicated that Fascin-1 knockdown inhibited TAZ from entering the nucleus of microglia. We further explored the regulatory effect of Fascin-1 on TAZ in microglia. Western blot results suggested that the expression of TAZ and Fascin-1 was significantly upregulated in microglia transfected with the Flag-tagged expression vector Flag-Fascin-1 compared with those transfected with the empty vector Flag (Figures 6E–G). Subsequently, Fascin-1 overexpression induced TAZ nuclear accumulation of microglia in comparison to that in the control group, as observed by immunofluorescence staining (Figure 6H). These results illustrated the relationship that Fascin-1 upregulated TAZ expression and mediated TAZ entry into the nucleus of microglia *in vitro*.

### The Interaction of Fascin-1 With Transcriptional Coactivator With PDZ-Binding Motif May Lead to Transcriptional Coactivator With PDZ-Binding Motif Nuclear Accumulation in Microglia

**FIGURE 7:**
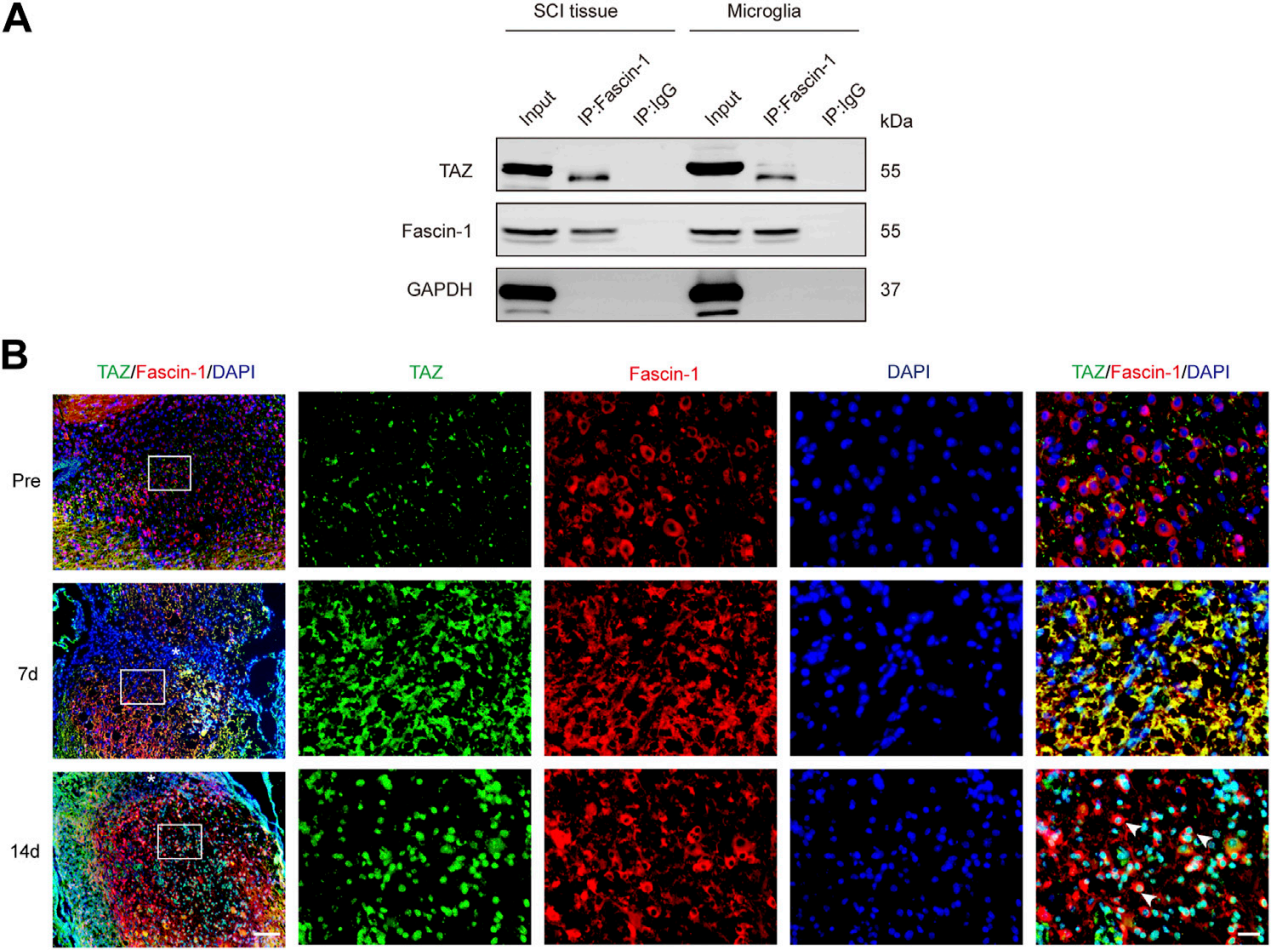
Fascin-1 interacted with TAZ and mediated the entry of TAZ into the nuclei of microglia *in vivo*. **(A)** Coimmunoprecipitation (Co-IP) analysis of the interaction between Fascin-1 and TAZ in myelin-treated microglia and 7 days SCI tissues. IP, immunoprecipitation; Input, total cells and tissue lysate; WB, Western blot analysis. IgG was used as a control IP. Pull down of the endogenous Fascin-1 protein complex was performed using mouse monoclonal Fascin-1 antibody. Fascin-1 and TAZ proteins were detected after Western blot the resulting immunoprecipitates using the rabbit polyclonal Fascin-1 antibody, rabbit monoclonal TAZ antibody. **(B)** Immunofluorescence markers from the sagittal section of the spinal cords showed the spatio-temporal distribution of Fascin-1 (red), TAZ (green), and DAPI (blue) at pre, 7 and 14 days after SCI. White arrows indicated that TAZ accumulated the nuclei of Fascin-1^+^ microglia. The asterisks indicated the centre of the lesion. Scale bars: low magnification, 100 μM; higher magnification, 20μM.

It has been previously shown that Fascin-1 can regulate TAZ, conversely, TAZ can also regulate Fascin-1 expression (Lim, Park et al., 2014; Kang, Wang et al., 2018), suggesting mutual regulation between Fascin-1 and TAZ, and therefore we further examined the interaction between Fascin-1 and TAZ in microglia and SCI. The endogenous interaction of Fascin-1 and TAZ was demonstrated by Co-IP assays in the spinal cord at 7 days after SCI and in myelin-treated microglia, which have been widely used *in vitro* to simulate the microenvironment of SCI (Yu, Cheng et al., 2021). The results showed that the anti-Fascin-1 antibody specifically coprecipitated TAZ, indicating that Fascin-1 could interact with TAZ after SCI (Figure 7A). Subsequently, we tested the changes in the distribution of Fascin-1 and TAZ in spinal cord tissue on different days after SCI. At 7 days, we observed that Fascin-1 colocalized with TAZ in the cytoplasm of microglia. Interestingly, at 14 days, Fascin-1 was expressed in the cytoplasm of microglia, while TAZ displayed a nuclear distribution in microglia (Figure 7B). Taken together, these results revealed that the interaction of Fascin-1 with TAZ may lead to TAZ nuclear accumulation in microglia after SCI.

### Transcriptional Coactivator With PDZ-Binding Motif is Downstream of Fascin-1 in the Regulation of Microglial Migration

**FIGURE 8:**
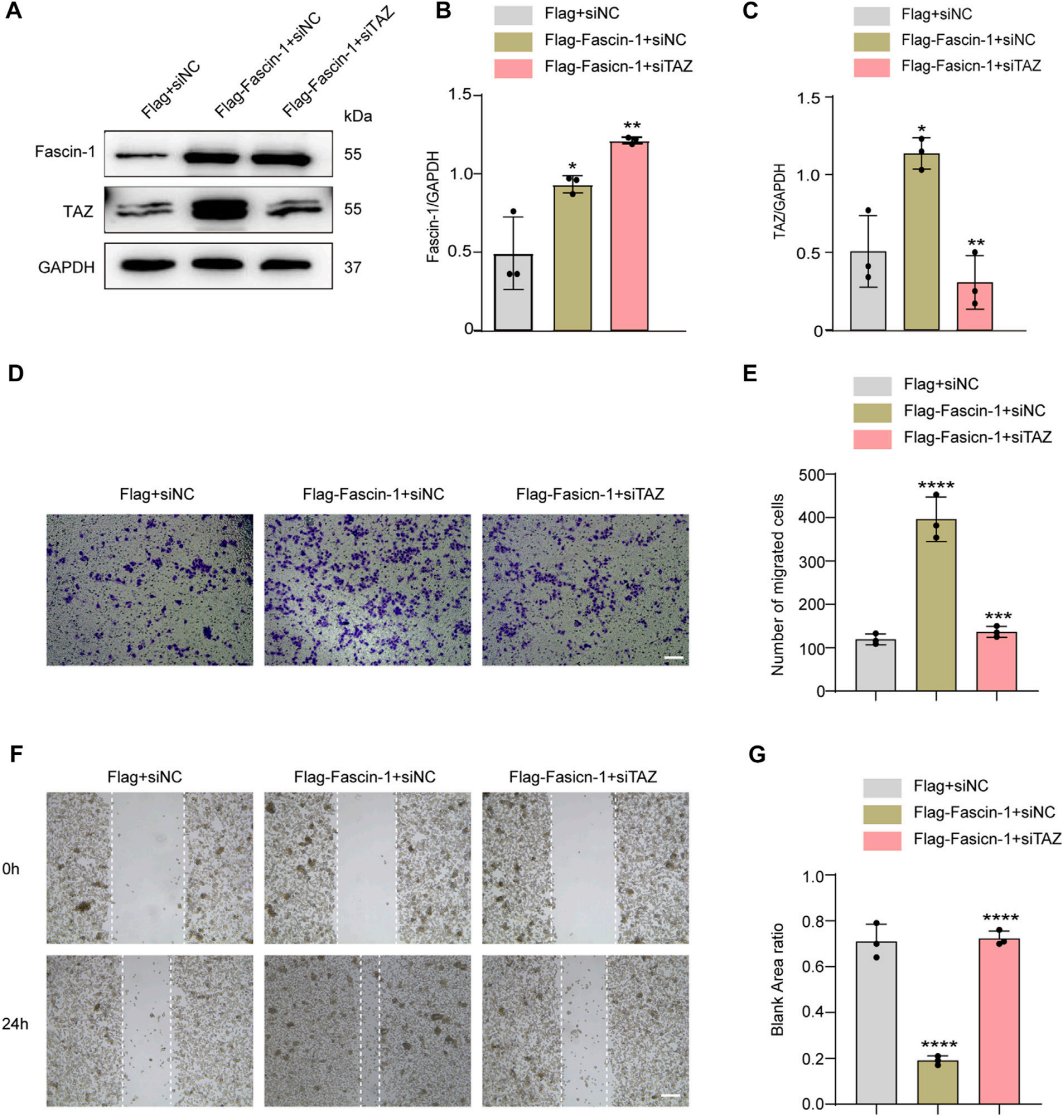
TAZ was downstream of Fascin-1 for regulating microglial migration. **(A)** Western blot was used to detect protein expression changes of Fascin-1 and TAZ after different treatment groups, including Flag+siNC, Flag-Fascin-1+siNC, Flag-Fascin-1+siTAZ (*n* = 3 per group). **(B,C)** Quantitative analysis of the relative levels of Fascin-1 (B) and TAZ (C) as shown in (A). The protein expression was normalized to GAPDH. Data were mean ± SEM. *p<0.05 (Flag-Fascin-1+siNC vs Flag+siNC); **p<0.01 (Flag-Fascin-1+siTAZ vs Flag-Fascin-1+siNC) in (B and C). **(D,F)** Scratch and Transwell tests were used to detect microglial migration in the above treatment groups (*n* = 3 per group). **(E)** Quantitative analysis of the number of transmembrane cells in (D). Data were mean ± SEM. **p<0.01 (Flag-Fascin-1+siNC vs. Flag+siNC), **p<0.01 (Flag-Fascin-1+siTAZ vs. Flag-Fascin-1+siNC). Scale bar: 200 μM. **(G)** Quantification of blank area ratio in (F). Data were mean ± SEM. ****p<0.0001 (Flag-Fascin-1+siNC vs. Flag+siNC), ****p<0.0001 (Flag-Fascin-1+siTAZ vs. Flag-Fascin-1+siNC). Scale bar: 200 μM.

The above data confirmed that TAZ was involved in the migration of microglia. Fascin-1 upregulated TAZ expression, induced TAZ nuclear accumulation in microglia, and interacted with TAZ in microglia. Next, we clarified the effect of Fascin-1 regulation of TAZ on microglial migration. We designed a rescue experiment, in which microglia were transfected with Flag+siNC, Flag-Fascin-1+siNC and Flag-Fascin-1+siTAZ. Western blot analysis showed that Fascin-1 overexpression enhanced the expression of Fascin-1 and TAZ, simultaneous Fascin-1 overexpression and TAZ knockdown reversed the upregulated the expression of TAZ caused by Fascin-1 overexpression (Figures 8A–C). The results of Transwell and scratch assays showed that Fascin-1 overexpression significantly promoted the migration of microglia, compared with that of the control group. Moreover, simultaneous Fascin-1 overexpression and TAZ knockdown reversed the enhanced microglial migration caused by Fascin-1 overexpression (Figures 8D–G). These results demonstrated that TAZ was a downstream molecule of Fascin-1 for regulating microglial migration.

## Discussion

**FIGURE 9:**
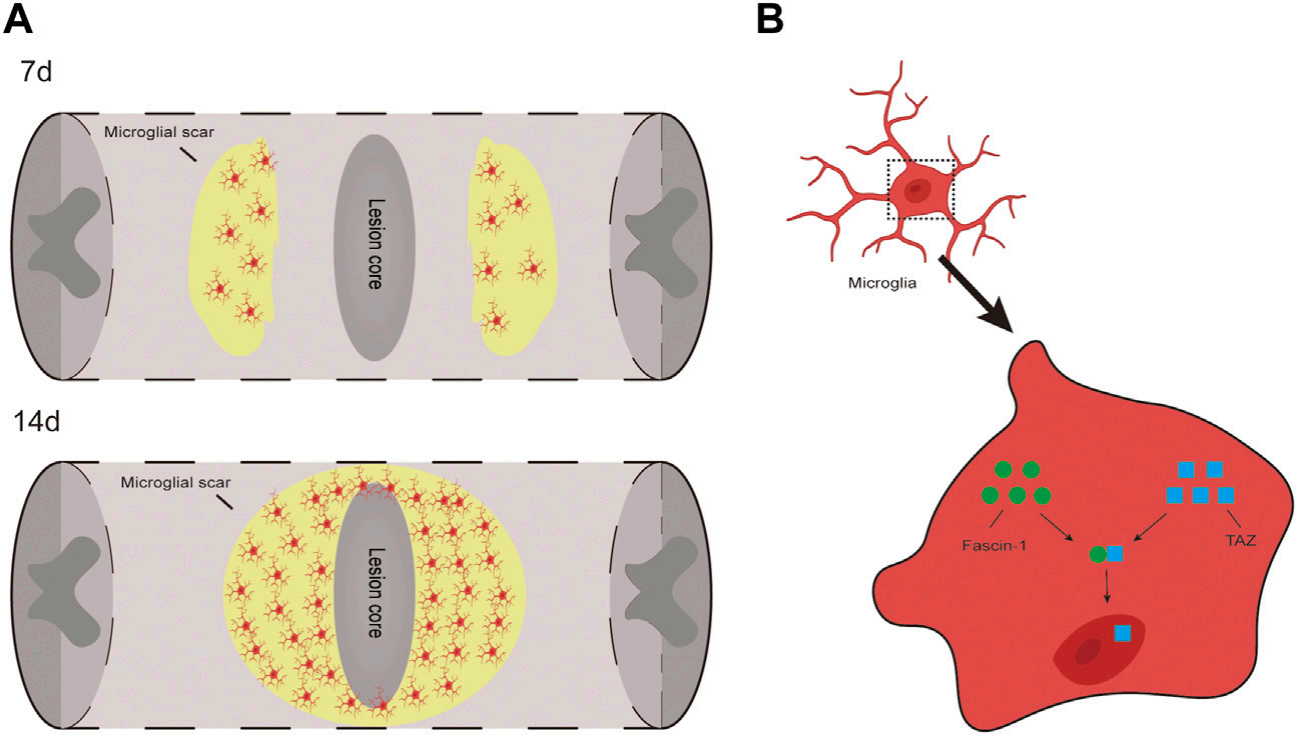
TAZ can promote the migration and accumulation of microglia around the lession core to form microglial scar after SCI and Fascin-1-TAZ pathway axis for positively regulating the migration of microglia. In this model, TAZ was downstream of Fascin-1 for regulating microglial migration. TAZ promoted the migration of microglia by Fascin-1 inducing TAZ nuclear accumulation of microglia, which contributed to the migration and accumulation of microglia around the lesion border to form microglial scars and promote functional recovery after SCI.

In this study, we present three important findings. First, we demonstrated that the TAZ protein was upregulated and displayed nuclear distribution in microglia 14 days after SCI. Additionally, TAZ was upregulated and entered the nucleus in M2-L microglia or myelin-treated microglia. Second, XMU-MP-1 (an inhibitor of Hippo kinase MST1/2 to activate TAZ) facilitated the formation of microglial scars and functional recovery following SCI, and TAZ promoted the microglial migration *in vitro*. Third, Fascin-1 interacted with TAZ, and Fascin-1 upregulated TAZ and mediated TAZ entry into the nucleus. We demonstrated that TAZ was a downstream molecule of Fascin-1 that regulates microglial migration to form microglial scars around the lesion core after SCI (Figure 9).

Glial scars are dense barriers formed by proliferating scar-forming microglia, which are essential for repair and recovery injuries in the central nervous system (CNS) (Tran, Warren et al., 2018). Glial scars impede the diffusion of inflammation, and stimulate vascular remodelling to heighten nutritional and metabolic support to the nervous tissue and promote the recovery of neurological functions (Stichel and Müller 1998). Microglia that migrate to the lesion edge cause secondary damage by secreting proinflammatory cytokines, reactive oxygen species, and proteases (Xu, Wang et al., 2021). However, a recent study revealed that scar forming microglia efficiently facilitated the neurological function recovery (Qian, Kong et al., 2022). This finding suggested that the formation of microglial scars may facilitate neurological function recovery after SCI. Consistently, in this study, we found that promoting the formation of glial scars by activating microglial TAZ improved behavioural recovery after SCI. Interestingly, our data revealed that TAZ was upregulated and activated in microglia after SCI, which was consistent with a previous report that revealed that TAZ was mostly found in secondary neurons and microglia of the spinal cord (Li, Lim et al., 2013). Therefore, we speculated that microglial TAZ may promote functional recovery by promoting microglial migration to affect the formation of glial scars after SCI.

Microglia are deemed one of the earliest and most crucial participants in neuroinflammation of the CNS, having harmful or advantageous influences on remyelination and neural regeneration (Colonna and Butovsky 2017). Following SCI, microglia initially differentiate into the M1-L and M2-L phenotypes, which are essential for regulating the microenvironment of SCI (Han, Yu et al., 2018). M1-L microglia aggravate neuroinflammation, in contrast, M2-L microglia facilitate tissue repair and play a crucial role in anti-inflammatory processes and the removal of cellular debris (Miron, Boyd et al., 2013). Unlike other tissues that possess self-generative ability, M2-L microglia do not persist for a long time following SCI, and M1-L microglia ultimately predominate over M2-L microglia (Kigerl, Gensel et al., 2009). Our study revealed that TAZ was upregulated and accumulated in the nuclei of M2-L microglia *in vitro*. However, more research is needed to see if there is a link between microglial polarization and TAZ expression *in vivo*.

Neural repair within the CNS has been extremely challenging because of the limited abilities of neurons to regenerate, particularly in a harmful inflammatory microenvironment that is also filled with myelin debris, therefore, clearance of damaged myelin debris is essential for improving CNS repair (Miron, Boyd et al., 2013). Myelin debris is a hallmark of SCI, and not only includes suppressive molecules that hinder remyelination and axon regeneration, but can also induce inflammatory responses (Kopper and Gensel 2018). Microglia are crucially involved in myelin uptake at demyelinated lesions (Neumann, Kotter et al., 2009). It has been reported that microglia tend to alter their phenotype towards the M2-L phenotype during the process of myelin debris clearance (Miron, Boyd et al., 2013). Consistently, our data suggested that TAZ was upregulated and accumulated in the nucleus in myelin-treated microglia *in vitro*, and the results were consistent with those of TAZ in M2-L microglia. However, the relationship between microglial phagocytosis of myelin and TAZ expression *in vivo* needs to be further clarified*.*

A recent report suggested that the Hippo signalling pathway was crucial to neuronal development and diseases (Emoto 2011). The key elements of the Hippo pathway, such as MST1 and large tumour suppressor 1 (LATS1), participate in neural regeneration and functional recovery. MST1 is an the upstream suppressor protein whose deficiency promotes spinal motor neuron survival by heightening autophagic flux (Zhang, Tao et al., 2017). Furthermore, suppression of LATS1 facilitated astrocyte proliferation after SCI (Wang and Chen 2018). YAP/TAZ are the core components of the Hippo pathway and regulate related target genes for tumour invasion and metastasis (Jeong, Kim et al., 2021). YAP mainly promoted astrocytic proliferation after SCI (Xie, Shen et al., 2020). This finding indicated that TAZ may participate in microglial migration after SCI. Our results confirmed that TAZ promoted microglial migration *in vitro.* Moreover, treatment with XMU-MP-1 (an inhibitor of Hippo kinase MST1/2 to activate TAZ) facilitated the migration and accumulation of microglia to form microglial scars at the lesion border and improved the functional recovery of mice after SCI, which was consistent with other studies reporting that TAZ can promote the migration of cells (Jeong, Kim et al., 2021). However, the specific effect of TAZ on functional recovery and the formation of microglial scars following SCI should be further confirmed utilizing microglial TAZ-conditional knockout (CKO) transgenic mice.

Fascin-1 is a cytoskeleton-organizing protein that induces membrane protrusions and cell migration by binding to core actin bundles inside microvillar projections and filopodia extensions (Sun, He et al., 2011). Recent reports have shown that Fascin-1 plays an important role in the migratory and intrusiveness of tumour cells through the Hippo pathway (Tong, Yee et al., 2005). YAP and its paralogue TAZ are downstream effectors of the Hippo pathway, and the functional role of TAZ is similar to that of YAP, as YAP and TAZ are negatively regulated by the Hippo signalling pathway (Jeong, Kim et al., 2021). It has been reported that YAP and TAZ can be regulated via Fascin-1. For instance, Fascin-1 promotes liver cancer cell migration and invasion by decreasing YAP and LATS1 phosphorylation and raising total and nuclear levels of YAP activation in liver cancer cells (Pu, Huang et al., 2021). A previous study showed that YAP was elevated and activated in astrocytes after SCI, whereas, Fascin-1 was specifically expressed in microglia and regulated the migration of microglia after SCI (Xie, Shen et al., 2020; Yu, Cheng et al., 2021). Hence, the Fascin-1-YAP pathway cannot facilitate microglial migration after SCI. Furthermore, increasing evidence suggests that the Hippo/TAZ signalling pathway is involved in cancer cell migration, invasion and actin cytoskeleton remodeling (Jeong, Kim et al., 2021). This finding implied that Fascin-1 activity in microglial migration could mediate Hippo/TAZ after SCI. Consistently, we confirmed that TAZ was highly expressed in microglia and that Fascin-1 upregulated the expression of TAZ to promote microglial migration. Additionally, our data indicated that TAZ may enter the nucleus of microglia after the interaction between Fascin-1 and TAZ and that interaction of Fascin-1 with TAZ may lead to TAZ nuclear accumulation in microglia after SCI. Unfortunately, the underlying mechanism of the influence of Fascin-1 on TAZ in the SCI microenvironment, and the interaction domain between Fascin-1 and TAZ need to be further investigated.

In summary, our study revealed that TAZ was elevated and activated in microglia after SCI. Functionally, TAZ may promote the migration and accumulation of microglia around the injury edge to form microglial scars and the recovery of neurological function after SCI. Mechanistically, TAZ was downstream of Fascin-1 in the positive regulation of microglial migration. Therefore, clarifying this mechanism will provide new ideas for the treatment of SCI.

## Data Availability

The original contributions presented in the study are included in the article/supplementary material, further inquiries can be directed to the corresponding authors.
