# Corrigendum: TAZ induces migration of microglia and promotes neurological recovery after spinal cord injury

**DOI:** 10.3389/fphar.2022.995767

**Published:** 2022-09-09

**Authors:** Xuyang Hu, Jinxin Huang, Yiteng Li, Lei Dong, Yihao Chen, Fangru Ouyang, Jianjian Li, Ziyu Li, Juehua Jing, Li Cheng

**Affiliations:** Department of Orthopaedics, The Second Hospital of Anhui Medical University, Hefei, China

**Keywords:** TAZ, Fascin-1, microglia, migration, spinal cord injury

In the published article, there were errors in “Figure 5 and Figure 8” as published. Due to processing a large number of images in different groups at one time, certain images were mixed between groups, resulting in the unintentional misplacement of the representative images in Figures 5C,K and Figures 8D,F. The authors provided the journal with the original data files. The corrected Figure 5 and Figure 8 appear below. The authors have checked the original images and adjusted the statistical analysis, and there is no problem with the scientific conclusions.

**FIGURE 5:**
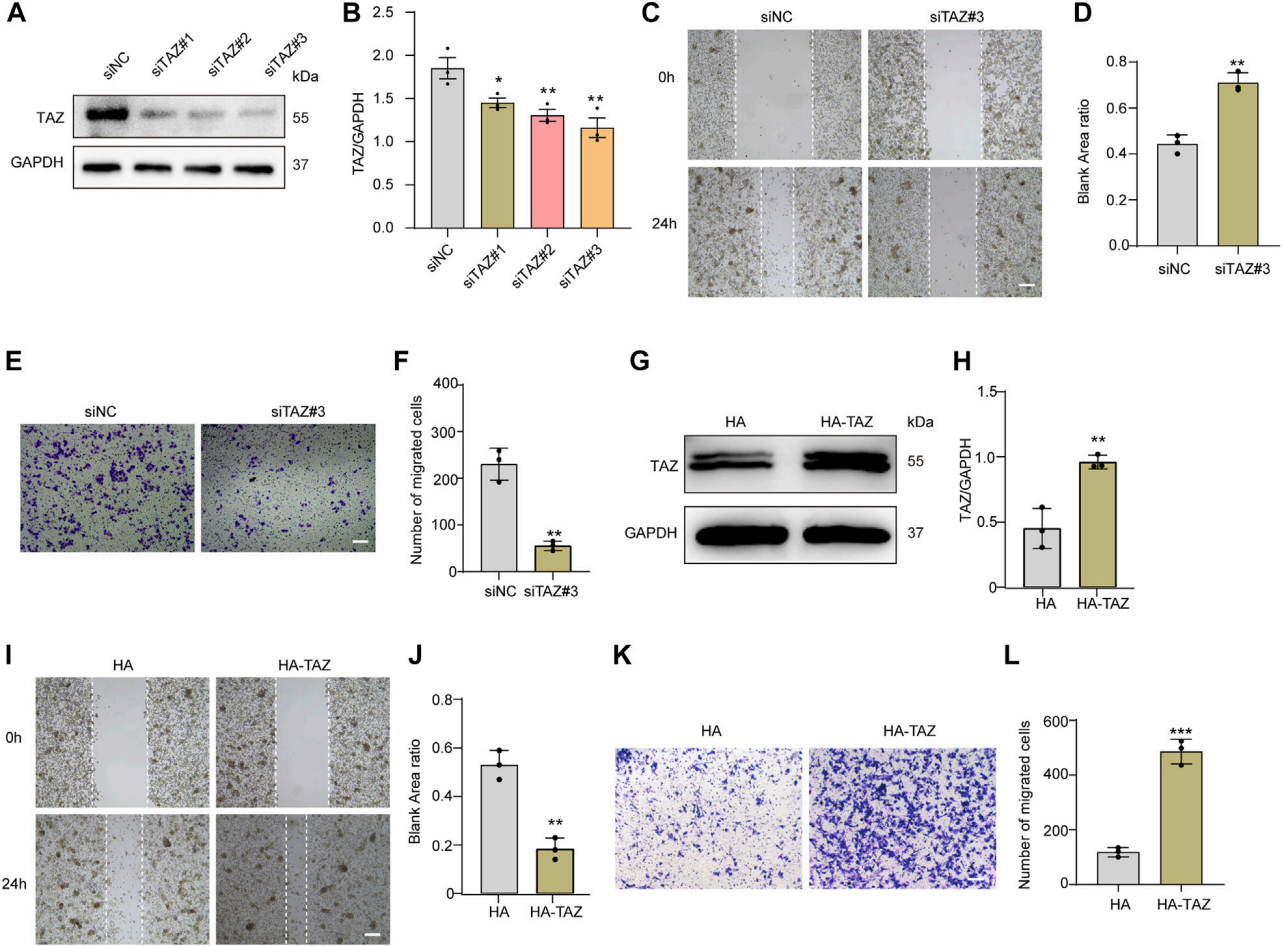
TAZ promoted microglial migration *in vitro*. **(A)** Western blot analysis of the expression of TAZ was detected after transfection with siTAZ (knockdown) and siNC (control). **(B)** Quantitative analysis of TAZ expression in **(A)**. GAPDH was used as the loading control. The blots (*n* = 3 per group) were quantified as previously described. Data were mean ± SEM. *p < 0.05 (siNC vs. siTAZ#1); **p < 0.01 (siNC vs. siTAZ#2 or siTAZ#3). According to the results, siTAZ#3 knockdown was the best effect on transfection, and siTAZ#3 was selected for transfection. **(C,E)** For the scratch assay and Transwell analysis were used to detect the migration of microglia after transfection with siNC and siTAZ#3 for 24 h, and cell migration was recorded 0 and 24 h (*n* = 3 per group). Scale bar: 200 μm. **(D)** Quantification of blank area ratio in **(C)**. Data were mean ± SEM. **p < 0.01 (siNC vs. siTAZ). **(F)** Quantitative analysis of the number of transmembrane cells in **(E)**. Data were mean ± SEM. **p < 0.01 (siNC vs. siTAZ). Scale bar: 200 μm. **(G)** Western blot analysis of the expression of TAZ was detected after plasmid HA-TAZ (overexpression) and HA (control) were transfected into microglia. **(H)** Quantitative analysis of TAZ expression in **(G)**. The imprints (*n* = 3 per group) were quantified by densitometry using ImageJ software. Data were mean ± SEM. **p < 0.01 (HA vs. HA-TAZ). **(I,K)** Scratch and Transwell analysis, which were used to detect microglial migration after transfection with plasmid HA-TAZ and HA into microglia for 24 h (*n* = 3 per group), cell migration was recorded 0 and 24 h. Scale bar: 200 μm. **(J)** Quantification of blank area ratio in **(I)**. Data were mean ± SEM. **p < 0.01 (HA vs. HA-TAZ). **(L)** Quantitative analysis of the number of transmembrane cells in **(K)**. Data were mean ± SEM. ***p < 0.001 (HA vs. HA-TAZ). Scale bar: 200 μm.

**FIGURE 8:**
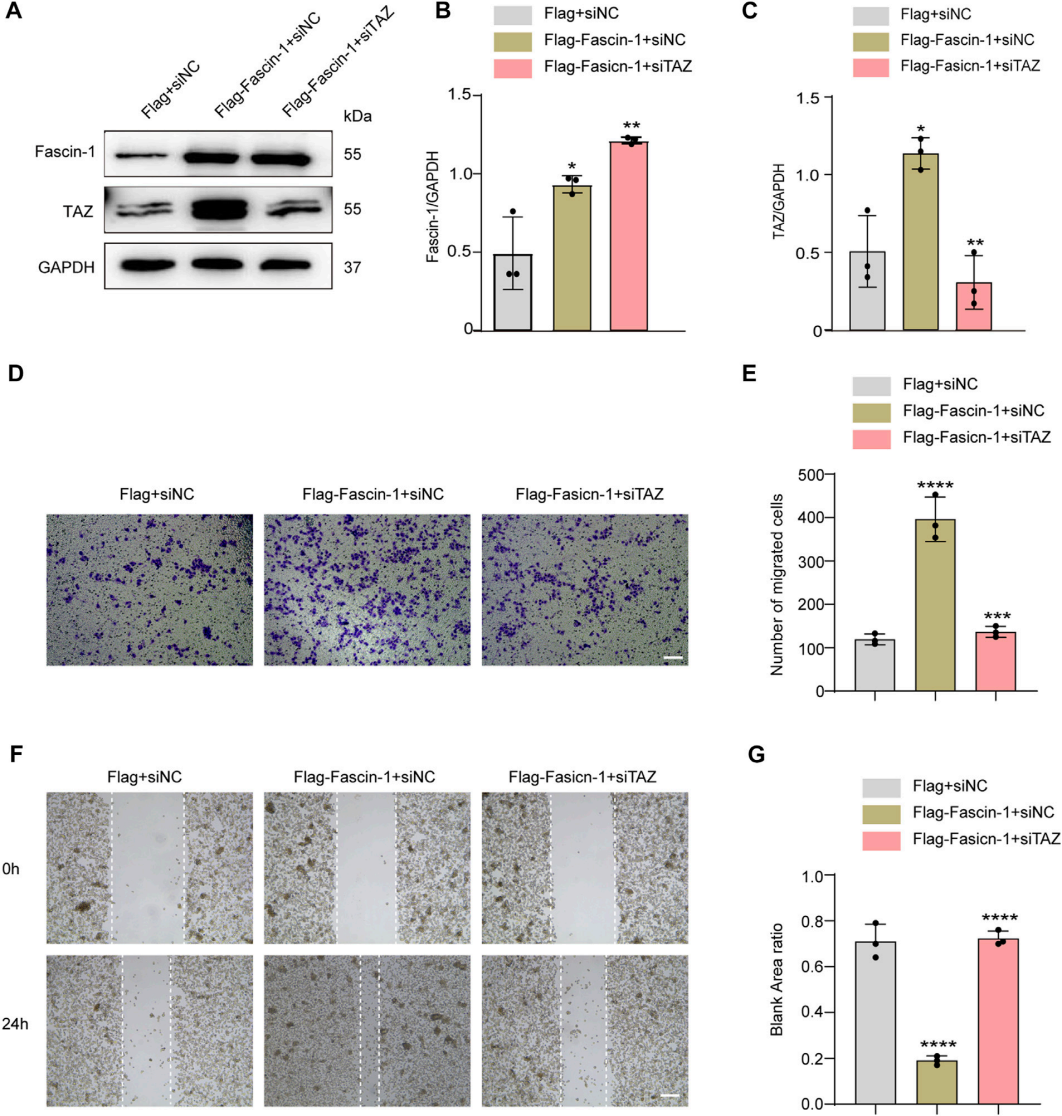
TAZ was downstream of Fascin-1 for regulating microglial migration. **(A)** Western blot was used to detect protein expression changes of Fascin-1 and TAZ after different treatment groups, including Flag+siNC, Flag-Fascin-1+siNC, Flag-Fascin-1+siTAZ (*n* = 3 per group). **(B,C)** Quantitative analysis of the relative levels of Fascin-1 **(B)** and TAZ **(C)** as shown in **(A)**. The protein expression was normalized to GAPDH. Data were mean ± SEM. *p < 0.05 (Flag-Fascin-1+siNC vs. Flag+siNC); **p < 0.01 (Flag-Fascin-1+siTAZ vs. Flag-Fascin-1+siNC) in **(B,C)**. **(D,F)** Scratch and Transwell tests were used to detect microglial migration in the above treatment groups (*n* = 3 per group). **(E)** Quantitative analysis of the number of transmembrane cells in **(D)**. Data were mean ± SEM. ****p < 0.0001 (Flag-Fascin-1+siNC vs. Flag+siNC), ***p < 0.001 (Flag-Fascin-1+siTAZ vs. Flag-Fascin-1+siNC). Scale bar: 200 μm. **(G)** Quantification of blank area ratio in **(F)**. Data were mean ± SEM. ****p < 0.0001 (Flag-Fascin-1+siNC vs. Flag+siNC), ****p < 0.0001 (Flag-Fascin-1+siTAZ vs. Flag-Fascin-1+siNC). Scale bar: 200 μm.

The authors apologize for this error and state that this does not change the scientific conclusions of the article in any way. The original article has been updated.

